# Survey of Ebola Viruses in Frugivorous and Insectivorous Bats in Guinea, Cameroon, and the Democratic Republic of the Congo, 2015–2017

**DOI:** 10.3201/eid2412.180740

**Published:** 2018-12

**Authors:** Helene M. De Nys, Placide Mbala Kingebeni, Alpha K. Keita, Christelle Butel, Guillaume Thaurignac, Christian-Julian Villabona-Arenas, Thomas Lemarcis, Mare Geraerts, Nicole Vidal, Amandine Esteban, Mathieu Bourgarel, François Roger, Fabian Leendertz, Ramadan Diallo, Simon-Pierre Ndimbo-Kumugo, Justus Nsio-Mbeta, Nikki Tagg, Lamine Koivogui, Abdoulaye Toure, Eric Delaporte, Steve Ahuka-Mundeke, Jean-Jacques Muyembe Tamfum, Eitel Mpoudi-Ngole, Ahidjo Ayouba, Martine Peeters

**Affiliations:** ASTRE of Centre de coopération internationale en recherche agronomique pour le développement, Institut national de la recherche agronomique and Univerisity of Montpellier, Montpellier, France (H.M. De Nys, P. Mbala Kingebeni, A.K. Keita, C. Butel, G. Thaurignac, C.-J. Villabona-Arenas, T. Lemarcis, M. Geraerts, N. Vidal, A. Esteban, M. Bourgarel, F. Roger, A. Toure, E. Delaporte, A. Ayouba, M. Peeters);; National Institute of Biomedical Research, Kinshasa, Democratic Republic of the Congo (P. Mbala Kingebeni, S.-P. Ndimbo-Kumugo, S. Ahuka-Mundeke, J.-J. Muyembe Tamfum);; Cliniques Universitaires de Kinshasa, Kinshasa (P. Mbala Kingebeni, S. Ahuka-Mundeke, J.-J. Muyembe Tamfum);; Centre de Recherche et de Formation en Infectiologie de Guinée, Conakry, Guinea (A.K. Keita, A. Toure);; Robert Koch-Institute, Berlin, Germany (F. Leendertz);; Ministère de l’Elevage et des Productions Animales, Conakry (R. Diallo);; Direction de Lutte contre la Maladie, Kinshasa (J. Nsio-Mbeta);; Royal Zoological Society of Antwerp, Antwerp, Belgium (N. Tagg);; Université de Conakry, Conakry (L. Koivogui);; Institut National de Sante Publique, Conakry (A. Toure);; Institut de Recherches Médicales et d’Études des Plantes Médicinales, Yaoundé, Cameroon (E. Mpoudi-Ngole);; Cameroon Institut de Recherche pout le Développement, Yaoundé (E. Mpoudi-Ngole)

**Keywords:** Ebola, bats, Africa, Guinea, Cameroon, the Democratic Republic of the Congo, frugivorous bats, insectivorous bats, Ebola virus, Zaire strain, Sudan strain, viruses, zoonoses, serology, seroprevalence, survey, ecology, Ebola virus infection, EVD, Ebola virus disease, cross-reactivity, Luminex, cutoff, *Mops* sp., *Eidolon helvum*, *Epomophorus gambianus*, *Hypsignathus monstrosus*, *Lissonycteris angolensis*, *Micropteropus pusillus*, *Rousettus aegyptiacus*

## Abstract

To clarify the role of bats in the ecology of Ebola viruses, we assessed the prevalence of Ebola virus antibodies in a large-scale sample of bats collected during 2015–2017 from countries in Africa that have had previous Ebola outbreaks (Guinea, the Democratic Republic of the Congo) or are at high risk for outbreaks (Cameroon). We analyzed 4,022 blood samples of bats from >12 frugivorous and 27 insectivorous species; 2–37 (0.05%–0.92%) bats were seropositive for Zaire and 0–30 (0%–0.75%) bats for Sudan Ebola viruses. We observed Ebola virus antibodies in 1 insectivorous bat genus and 6 frugivorous bat species. Certain bat species widespread across Africa had serologic evidence of Zaire and Sudan Ebola viruses. No viral RNA was detected in the subset of samples tested (n = 665). Ongoing surveillance of bats and other potential animal reservoirs are required to predict and prepare for future outbreaks.

Since the first outbreak of Ebola virus disease (EVD) in 1976 in the northern part of the Democratic Republic of the Congo (DRC), 26 recognized outbreaks have occurred in humans across Africa; fatality rates of outbreaks have been 25%–90% (*1**–**4*). Each EVD outbreak most likely resulted from independent zoonotic events.

Bats are believed to play a role in the ecology of Ebola viruses as a reservoir species (*5*). Bats might infect humans directly or via intermediate amplifying hosts, like nonhuman primates or duikers (*6**,**7*). Bats might serve as a source of infection in certain areas where bats are hunted and eaten as bushmeat, but infection could also occur after consumption of fruits contaminated with saliva, urine, or feces from Ebola virus–infected bats (*8**,**9*). Ebola virus emergence through exposure to bats was suspected for at least 2 outbreaks: Luebo (the DRC) in 2007 and West Africa in 2013 (*10**,**11*).

Relatively few data are available to support the role of bats in the ecology of Ebola viruses. During the EVD outbreaks of 2003 in Gabon and the Congo, Zaire Ebola virus RNA and antibodies were detected in live-caught specimens from 3 fruit bat species (*Epomops franqueti*, *Hypsignathus monstrosus*, *Myonycteris torquata*); virus sequences were found in the livers or spleens of a few bats (*6*). In subsequent studies in Gabon, the Congo, Ghana, and Zambia, antibodies were detected in additional frugivorous bat species (*Eidolon helvum*, *Epomophorus gambianus*, *Rousettus aegyptiacus*, *Micropteropus pusillus*) and 1 insectivorous species (*Mops condylurus*) (*12*–*16*). The amplification and sequencing of viral RNA of other filoviruses in bats, such as Marburg virus in bats from Africa (*17**–**20*), Lloviu virus in bats from Europe (*21*), and new filoviruses in bats from China (*22*), has provided additional evidence for a possible role of bats in Ebola virus ecology.

In general, EVD outbreaks have been limited in terms of their geographic spread and chains of human-to-human transmission (*1*). However, during the 2013–2016 outbreak, virus spread to the urban areas of 3 countries, infecting ≈30,000 persons in Guinea, Sierra Leone, and Liberia, and ≈11,000 deaths were recorded (*23*). This outbreak illustrated the potential for epidemic spread from a single zoonotic transmission, with severe public health and socioeconomic impact (*24*). Additional studies are urgently needed to identify the animal reservoir, predict EVD outbreak risks, and improve our capacity to control epidemics.

In previous modeling studies, areas were defined as at risk for EVD outbreaks on the basis of data collected from a limited number of wildlife bat species from a few geographic regions (*5*,*25*). Also, a wide variety of serologic assays and interpretation criteria have been used, making comparison of results challenging (*12*–*16*,*26*,*27*). For this study, we performed a large serosurvey with a highly specific and sensitive high-throughput assay to assess Ebola virus prevalence in bats from Africa (*28*). We studied bats from Guinea and the DRC, countries with previous EVD outbreaks, and Cameroon, a country considered at high risk for future EVD outbreaks (*5*,*25*).

## Materials and Methods

### Study Sites and Sample Collection

During November 2015–August 2017, we collected samples from free-ranging frugivorous and insectivorous bats in Guinea, Cameroon, and the DRC. We captured bats at night using ground mist nets or harp traps in roosting and foraging sites. We set up ground mist nets (12 × 3.2 m) of 30-mm and 60-mm mesh sizes at different heights (1–7 m) to maximize capture of different species. We opened nets or harp traps just before sunset and checked for bats every 1–2 hours. Captured bats were released the same night immediately after sampling. Using bat whole blood taken by venipuncture of the propatagial or brachial vein, we dropped blood samples directly onto Whatman 903 filter paper (GE Healthcare, Feasterville-Trevose, PA, USA). We air-dried and preserved samples individually in plastic bags containing silica desiccant and stored them in hermetic boxes; 2–3 weeks later, we transferred dried blood spots to −20°C until needed for analysis. Data recorded in the field included information on capture site (global positioning system coordinates, ecologic environment), capture method, morphology (body measurements, weight, color), sex, age class (adult, juvenile), and species (identified visually). We collected negative control samples (n = 145) from a captive-born insectivorous bat species (103 *Carollia perspicillata* bats) hosted at the Parc Zoologique de Montpellier (Montpellier, France) and 2 frugivorous bat species (19 *Pteropus giganteus* bats, 23 *R. aegyptiacus* bats) hosted at Wilhelma Zoo and Botanical Garden (Stuttgart, Germany). We collected and preserved samples the same way we did for free-ranging bats.

### Screening for Ebola Virus Antibodies

We tested dried blood spots with a Luminex-based serologic assay adapted for bats (*28*) (Technical Appendix). The assay included recombinant Ebola virus proteins glycoprotein, nucleoprotein, or viral protein 40 for different lineages: Zaire, Sudan, Bundibugyo, and Reston. We reconstituted plasma from dried blood spots as previously described (*28*) and incubated 100 μL of sample (final plasma dilution 1:2,000) with 50 µL of recombinant protein–coated beads (2 µg protein/1.25 × 10^6^ beads) in 96-well flat-bottom filter plates (Millipore, Tullagreen, Ireland) on a plate shaker at 300 rpm for 16 h at 4°C in the dark. After washing, we added 0.1 μg/mL of goat anti–bat biotin–labeled IgG (Euromedex, Souffelweyersheim, France) per well and incubated for 30 min at 300 rpm. After another round of washing, we added 50 µL of 4 µg/mL streptavidin-R-phycoerythrin (Fisher Scientific, Illkirch, France) per well and incubated for 10 min at 300 rpm. Reactions were read with BioPlex-200 (BioRad, Marnes-la-Coquette, France). We expressed results as median fluorescence intensity (MFI) per 100 beads. We included 3 samples on every plate to validate interassay repeatability.

### Determination of Cutoffs

In the absence of positive control samples, we used 4 different statistical methods to determine the MFI cutoff value for each antigen (*29*,*30*) (Technical Appendix Table 1). First, we used a general formula that involved the MFI of the 145 negative control samples, and we assigned the cutoff as mean plus 4 times the SD (mean + 4×SD). Second, we used a change point analysis (*31*) to identify the value at which statistical properties of the underlying probability distribution changed. This value was used to identify outliers and classify them as reactive. We used the R package changepoint (*32*) to calculate a single shift in the arithmetic mean with the at-most-1-change method (*33*). Third, we fitted univariate distributions to our data and defined the cutoff as a 0.001 risk for error, as was used in other virus serology studies (*13*,*34*). We reduced the set of candidate distributions following a bootstrapped skewness-kurtosis analysis (*35*). We performed fitting by maximum-likelihood estimation and selected the best-fit distribution on the basis of the Akaike information criteria with the R library fitdistrplus (*36*). A negative binomial distribution best-fit the data; however, we also used the negative exponential distribution as in Pourrut et al. and Laing et al. (*13*,*34*). For every antigen, we computed bootstrap values using 10,000 replicates and averaged. We performed analyses with R version 3.3.2 software (https://www.r-project.org/). We considered a blood sample reactive if the MFI of the reaction was above the cutoff. We defined Ebola virus antibody positivity as reactivity to glycoprotein and nucleoprotein of the same lineage, as was done in our previous study (*28*).

### Nucleic Acid Extraction and PCR Screening for Ebola Virus RNA

We extracted total DNA and RNA from dried blood spots as previously described using Nuclisens (bioMerieux, Marcy-l’Etoile, France) or m2000sp methods (Abbott Molecular Inc., Des Plaines, IL, USA), which are known for a high performance recovering nucleic acids from dried blood spots (*37*,*38*). For bat species from Cameroon and Guinea, we screened for Zaire Ebola virus RNA by seminested reverse transcription PCR (RT-PCR) targeting the nucleoprotein region of the virus genome. We amplified a 126-bp fragment of Zaire Ebola virus using primers NP1F1 (forward, 5′-CGGACACACAAAAAGAAWGAA-3′) and NP1R-ZR (reverse, 5′-CTCTATCTTKGTGATRTGGCTCTGA-3′) in the first round of PCR and NP1F2 (forward, 5′- TTGTGTGCGARTAACTAYGAGGAAG-3′) plus NP1R-ZR in the second round. For species from the DRC, we performed seminested RT-PCR targeting the viral protein 35 region of the genome using the protocol of He et al. with modifications (*41*). In the first round, we amplified a 217-bp fragment with primers VP35-F (5′-ATYATGTATGATCACYTVCCWGG-3′) and VP35-R (5′-AGCGRATGTGGATSACRGGT-3′) and, in the second round, a 184-bp product with primers VP35-R and VP35-in-F (5′-GCTTTYCAYCAAYTAGTRCAAG-3′).

### Molecular Confirmation of Bat Species

We confirmed bat species identification recorded in the field on a subset of samples by using molecular tests. We amplified an ≈800-bp fragment of mitochondrial cytochrome b using primers cytb-L14724 (forward) and cytb-H15506 (reverse) (*11*,*39*,*40*). We substituted the cytb-L14724 primer with cytb-L140217 (5′-ATGACCAACATCCGAAAATCNCAC-3′) to improve PCR performance for certain species. We purified PCR products through agarose gel (1%) and directly sequenced on an ABI 3500 sequencer (Applied Biosystems, Courtaboeuf, France). We performed BLAST analyses (https://blast.ncbi.nlm.nih.gov/Blast.cgi) to identify the most similar bat species. For samples with no or low similarity (<97%) hits with species in GenBank, we performed phylogenetic analyses with newly obtained sequences and reference sequences for different bat species using maximum-likelihood methods implemented with PhyML (http://www.atgc-montpellier.fr/phyml/) to determine genus.

## Results

### Bat Species and Sampling

**Figure 1:**
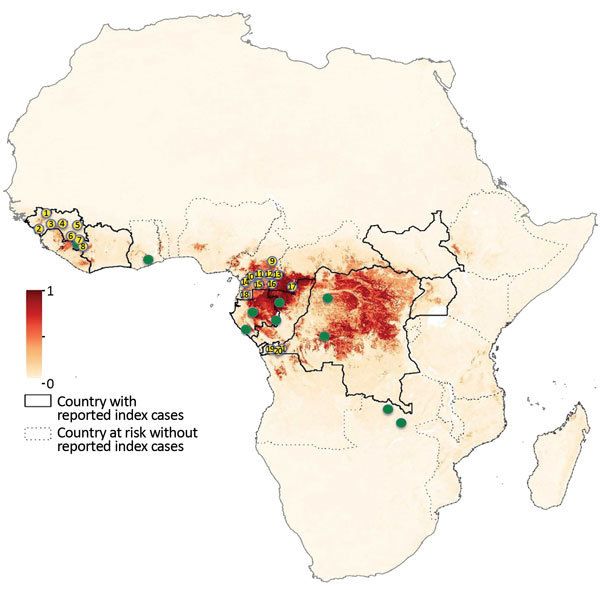
Study sites for bat blood sample collection for Ebola virus serology, Guinea, Cameroon, and the Democratic Republic of the Congo, 2015–2017. Yellow dots indicate sampling sites for bats in our study, and green dots indicate sampling sites in previously published studies. Dark red shading indicates highest and light yellow lowest risk for Ebola virus spillover events. Study sites are numbered: 1, Koundara; 2, Conakry; 3, Kindia; 4, Mamou; 5, Kankan; 6, Gueckedou; 7, Macenta; 8, Nzerekore; 9, Mbam Djerem; 10, Libellengoi Sud; 11, Yaoundé; 12, Ekom; 13, North Dja; 14, Bipindi; 15, Mbalmayo; 16, Djoum; 17, Mambele; 18, Campo M’an; 19, Boma; 20, Kimpese; 21, Zongo. Countries with reported index Ebola cases and countries without such cases but deemed at risk are indicated. Map of Africa adapted from Pigott et al. (*5*) (https://creativecommons.org/licenses/by/4.0/) by adding locations of collection sites.

We analyzed blood samples from 4,022 wild bats from 21 different regions in Cameroon (n = 10), Guinea (n = 8), and the DRC (n = 3) (Figure 1; Table 1). To increase species diversity, we captured bats in multiple ecologic settings: forests (49%), open fields (10%), villages (29%), plantations (7%), and urban areas (5%). For 1,470 (36.5%) samples, species identification in the field was confirmed by sequence analysis. At each site, >1 sample was confirmed per sampling date, capture method, and morphologic description. For the remaining samples, species identification was extrapolated by combining molecular and morphologic data, including photographs whenever available. For some insectivorous bat families (*Miniopteridae*, *Molossidae*, *Nycteridae*, *Rhinolophidae*), identification was possible only at the genus level; for some *Molossidae* bats, we could not distinguish between *Mops* and *Chaerephon* genera because of the lack of sequences in GenBank (Table 2). For 87 (2.16%) samples, species identification was not possible because incomplete data were recorded in the field, and available biologic materials were insufficient for molecular confirmation. We collected samples from 1,736 (43.2%) frugivorous bats (family *Pteropodidae*) of 12 species and 2,199 (54.7%) insectivorous bats (7 families) of >27 species. The insectivorous bat families sampled, in order of decreasing frequency, were *Hipposideridae* (31.9%), *Molossidae* (13.4%), *Miniopteridae* (5.8%), *Rhinolophidae* (2.1%), *Vespertilionidae* (0.8%), *Nycteridae* (0.5%), and *Emballonuridae* (0.12%). Overall, 54.7% of bats were female and 43.8% were male; for 1.5% (n = 60) of bats, sex was unknown. Most (77.9%) bats were adults, and 9.6% were juveniles; for 12.5% (n = 502) of bats, age could not be determined or was not recorded.

**Table 1:** Bat samples collected for Ebola virus serology by study site, Guinea, Cameroon, and the Democratic Republic of the Congo, 2015–2017

Country, site	No. samples
Democratic Republic of the Congo	
Boma	156
Kimpese	202
Zongo	472
Subtotal	830
Cameroon	
Yaoundé	126
Libellengoi Sud	44
Mbalmayo	48
Bipindi	479
Campo M’an	344
North Dja	295
Ekom	122
Djoum	56
Mambele	348
Mbam Djerem	156
Subtotal	2,018
Guinea	
Conakry	107
Kindia	323
Kankan	378
Koundara	90
Mamou	147
Gueckedou	49
Macenta	9
Nzerekore	71
Subtotal	1,174
Total	4,022

**Table 2:** Bat species sampled for Ebola virus serology, Guinea, Cameroon, and the DRC, 2015–2017*

Family	Species	DRC, no.	Cameroon, no.	Guinea, no.	Total, no.
*Emballonuridae*	*Coleura afra*	0	5	0	5
*Hipposideridae*	*Hipposideros abae*	0	0	37	37
	*H. beatus*	0	4	0	4
	*H. cyclops*	0	14	0	14
	*H. fuliginosus*	0	2	0	2
	*H. gigas*	2	9	2	13
	*H. jonesi*	0	1	12	13
	*H. ruber/caffer*	127	807	237	1,171
	*Hipposideros* sp.	28	0	0	28
Subtotal		157	837	288	1,282
*Miniopteridae*	*Miniopterus* sp.	205	0	27	232
*Molossidae*	*Chaerephon* sp.	0	0	44	44
	*Mops condylurus*	0	0	110	110
	*Mops* sp.	0	256	0	256
	*Mops/Chaerephon* sp.	0	8	120	128
Subtotal		0	264	274	538
*Nycteridae *	*Nycteris* sp.	0	7	15	22
*Rhinolophidae*	*Rhinolophus alcyone*	0	16	0	16
	*R. darlingii*	3	0	0	3
	*R. fumigatus*	0	0	19	19
	*R. landeri*	0	0	6	6
	*Rhinolophus* sp.	3	38	1	42
Subtotal		6	54	26	86
*Vespertilionidae*	*Glauconycteris variegata*	0	3	0	3
	*Kerivoula* sp.	0	1	0	1
	*Myotis bocagii*	0	3	0	3
	*Neoromicia* sp.	0	5	0	5
	*Scotophilus leucogaster*	0	0	15	15
	*S. nigrita*	0	0	1	1
	*S. nux*	0	6	0	6
Subtotal		0	18	16	34
*Pteropodidae*	*Eidolon helvum*	305	158	17	480
	*Epomophorus gambianus*	0	0	191	191
	*Epomophorus wahlbergi*	0	16	0	16
	*Epomops buettikoferi*	0	0	4	4
	*Epomops franqueti*	20	256	0	276
	*Hypsignathus monstrosus*	1	176	8	185
	*Lissonycteris angolensis*	22	30	32	84
	*Megaloglossus woermanni*	1	19	0	20
	*Micropteropus pusillus*	44	2	18	64
	*Myonycteris torquata*	35	21	0	56
	*Rousettus aegyptiacus*	0	131	228	359
	*Scotonycteris zenkeri*	0	1	0	1
Subtotal		428	810	498	1,736
Inderminate species		34	23	30	87
Total		830	2,018	1,174	4,022

*DRC, the Democratic Republic of the Congo.

### Bats Antibodies against Different Ebola Virus Antigens

**Figure 2:**
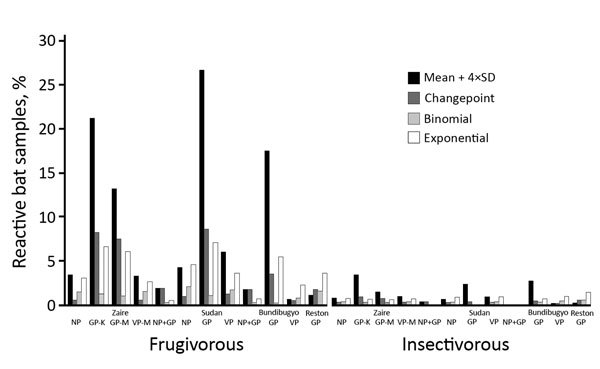
Bat blood samples reactive to Ebola virus antigens, by statistical method used to determine cutoff, Guinea, Cameroon, and the Democratic Republic of the Congo, 2015–2017. Samples from frugivorous bats (n = 1,736) and insectivorous bats (n = 2,199) were tested by Luminex assay with GP, NP, and VP of the Zaire and Sudan lineages; GP and VP of the Bundibugyo lineage; and GP of the Reston lineage. GP, glycoprotein; K, Kissoudougou strain; M, Mayinga strain; NP, nucleoprotein; VP, viral protein 40.

We tested all samples for Ebola virus antibodies. The number of samples reacting with >1 antigen was 734 (18.2%) by the mean + 4×SD method, 274 (6.8%) for the change-point method, 175 (4.4%) for the binomial method, and 457 (11.4%) for the exponential method. Blood samples frequently reacted with glycoprotein antigens; samples reacted most with Zaire and Sudan Ebola virus antigens and least with Reston (Table 3). Simultaneous reactivity to >1 antigen (i.e., glycoprotein, nucleoprotein, viral protein 40) from the same virus lineage was rare. Simultaneous reactivity to the same antigen from different virus lineages was frequent; 32.3%–76.7% of blood samples were reactive to glycoprotein from >2 Ebola virus species, 18.4%–34.0% to viral protein 40, and 1.5%–4.4% to nucleoprotein (Technical Appendix Table 2). When using the criterion simultaneous presence of antibodies to nucleoprotein and glycoprotein, the antibody positivity for Zaire or Sudan Ebola virus antibodies was generally <1% for all bats tested, regardless of cutoff method, and was lower among insectivorous than frugivorous bats: 0.05%–0.27% (insectivorous) and 0.06%–1.79% (frugivorous) for Zaire Ebola virus versus 0%–0.09% (insectivorous) and 0%−1.61% (frugivorous) for Sudan Ebola virus (Table 3; Figure 2). Three samples were positive for Zaire and Sudan Ebola viruses, but only by less stringent cutoff methods (i.e., mean + 4×SD).

**Table 3:** Blood samples from bats reactive with Ebola virus antigens in Luminex assay, by antigen, bat type, and statistical method used to determine cutoff, Guinea, Cameroon, and the Democratic Republic of the Congo, 2015–2017*

Ebola virus species, antigen	Bat type	Statistical method, no. (%)	Estimated range, %

Mean + 4×SD	Change point	Binomial	Exponential
Zaire						
NP	Frugivorous	57 (3.28)	8 (0.46)	24 (1.38)	51 (2.94)	0.46–3.28
NP	Insectivorous	15 (0.68)	1 (0.05)	6 (0.27)	15 (0.68)	0.05–0.68
NP	Total	72 (1.79)	9 (0.22)	30 (0.75)	66 (1.64)	0.23–1.79
GP-K	Frugivorous	365 (21.03)	141 (8.12)	20 (1.15)	113 (6.51)	1.15–21.03
GP-K	Insectivorous	73 (3.32)	18 (0.82)	2 (0.09)	12 (0.55)	0.09–3.32
GP-K	Total	440 (10.94)	160 (3.98)	22 (0.55)	125 (3.11)	0.55–10.94
GP-M	Frugivorous	226 (13.02)	128 (7.37)	16 (0.92)	103 (5.93)	0.92–13.02
GP-M	Insectivorous	31 (1.41)	14 (0.64)	2 (0.09)	12 (0.55)	0.09–1.41

GP-M	Total	259 (6.44)	143 (3.56)	18 (0.45)	115 (2.86)	0.45–6.44
VP	Frugivorous	55 (3.17)	8 (0.46)	24 (1.38)	44 (2.53)	0.46–3.17
VP	Insectivorous	19 (0.86)	5 (0.23)	6 (0.27)	14 (0.64)	0.23–0.86
VP	Total	75 (1.86)	14 (0.35)	30 (0.75)	59 (1.47)	0.35–1.86
NP + GP	Frugivorous	31 (1.79)	31 (1.79)	1 (0.06)	7 (0.40)	0.06–1.79
NP + GP	Insectivorous	6 (0.27)	6 (0.27)	1 (0.05)	1 (0.05)	0.05–0.27
NP + GP	Total	37 (0.92)	37 (0.92)	2 (0.05)	8 (0.20)	0.05–0.92
Sudan						
NP	Frugivorous	71 (4.09)	15 (0.86)	34 (1.96)	77 (4.44)	0.86–4.44
NP	Insectivorous	12 (0.55)	1 (0.05)	5 (0.23)	18 (0.82)	0.05–0.82
NP	Total	84 (2.09)	17 (0.42)	39 (0.97)	96 (2.39)	0.42–2.39
GP	Frugivorous	459 (26.44)	147 (8.47)	17 (0.98)	121 (6.97)	0.98–26.44
GP	Insectivorous	49 (2.23)	6 (0.27)	1 (0.05)	1 (0.05)	0.05–2.23
GP	Total	509 (12.66)	154 (3.83)	18 (0.45)	125 (3.11)	0.45–12.66
VP	Frugivorous	102 (5.88)	20 (1.15)	28 (1.61)	61 (3.51)	1.15–5.88
VP	Insectivorous	19 (0.86)	4 (0.18)	6 (0.27)	18 (0.82)	0.18–0.86
VP	Total	121 (3.01)	24 (0.60)	34 (0.85)	80 (1.99)	0.60–3.01
NP + GP	Frugivorous	28 (1.61)	28 (1.61)	0	10 (0.58)	0–1.61
NP + GP	Insectivorous	2 (0.09)	2 (0.09)	0	0	0–0.09
NP + GP	Total	30 (0.75)	30 (0.75)	0	10 (0.25)	0–0.75
Bundibugyo						
GP	Frugivorous	301 (17.34)	59 (3.40)	0	93 (5.36)	0–17.34
GP	Insectivorous	58 (2.64)	8 (0.36)	5 (0.23)	13 (0.59)	0.23–2.64
GP	Total	361 (8.98)	68 (1.69)	22 (0.55)	107 (2.66)	0.55–8.98
VP	Frugivorous	9 (0.52)	7 (0.40)	12 (0.69)	37 (2.13)	0.40–2.14
VP	Insectivorous	0	1 (0.05)	8 (0.36)	20 (0.91)	0–0.91
VP	Total	9 (0.22)	8 (0.20)	20 (0.5)	57 (1.42)	0.20–1.42
Reston						
GP	Frugivorous	17 (0.98)	28 (1.61)	26 (1.50)	61 (3.51)	0.98–3.51
GP	Insectivorous	3 (0.14)	10 (0.45)	10 (0.45)	29 (1.32)	0.14–1.32
GP	Total	20 (0.50)	38 (0.94)	36 (0.90)	90 (2.24)	0.50–2.24

*VP refers to viral protein 40 of Ebola virus. Results are presented for frugivorous (n = 1,736), insectivorous (n = 2,199), and total (n = 4,022) bats. GP, glycoprotein; K, Kissoudougou strain; M, Mayinga strain; NP, nucleoprotein; VP, viral protein.

### Zaire and Sudan Ebola Virus Reactivity of Different Bat Species

We estimated specific reactivity to Zaire and Sudan Ebola viruses by bat species. We did not include Bundibugyo and Reston because recombinant nucleoproteins were not available. Among insectivorous bats, only blood samples from *Mops* sp. bats (1–6/494) were positive for Zaire or Sudan Ebola virus antibodies (Table 4). Among frugivorous bats, samples from *E. helvum*, *H. monstrosus*, and *R. aegyptiacus* bats had the highest reactivity. We observed Zaire and Sudan Ebola virus seropositivity in these 3 species with almost all cutoff methods: 0.2%–3.3% for Zaire Ebola virus and 1.0%–2.9% for Sudan Ebola virus in *E. helvum* bat samples, 0.5%–1.6% for Zaire Ebola virus and 1.1%–4.3% for Sudan Ebola virus in *H. monstrosus* bat samples, and 0.6%–2.5% for Zaire Ebola virus and 0.8%–1.4% for Sudan Ebola virus in *R. aegyptiacus* bat samples. We observed 2.4% Zaire Ebola virus–seropositive samples for *Lissonycteris angolensis* bats and 0.5% for *Epomophorus* sp. bats, but only by less stringent cutoff methods. One sample from *M. pusillus* bats was seropositive for Sudan Ebola virus. No samples from *E. franqueti* or *M. torquata* bats were reactive with any Ebola virus antigens. Samples from the 1 *Scotonycteris zenkeri* bat and 20 *Megaloglossus woermanni* bats were seronegative. Overall, Zaire or Sudan Ebola virus antibodies were observed in 7 (1 insectivorous and 6 frugivorous) bat species.

**Table 4:** Blood samples from bats reactive with both nucleoprotein and glycoprotein of Zaire or Sudan Ebola virus, by statistical method used to determine cutoff, Guinea, Cameroon, and the Democratic Republic of the Congo, 2015–2017*

Bat family, genus	No. tested	Ebola virus species	Statistical method										
			Mean + 4×SD			Change-point			Binomial			Exponential	
			No.	% (95% CI)		No.	% (95% CI)		No.	% (95% CI)		No.	% (95% CI)
*Hipposideridae*													
* Hipposideros *sp.	1,282	Zaire	0	0 (0–0.3)		0	0 (0–0.3)		0	0 (0–0.3)		0	0 (0–0.3)
	1,282	Sudan	0	0 (0–0.3)		0	0 (0–0.3)		0	0 (0–0.3)		0	0 (0–0.3)
*Miniopteridae*													
* Miniopterus *sp.	232	Zaire	0	0 (0–1.6)		0	0 (0–1.6)		0	0 (0–1.6)		0	0 (0–1.6)
	232	Sudan	0	0 (0–1.6)		0	0 (0–1.6)		0	0 (0–1.6)		0	0 (0–1.6)
*Molossidae*													
* Chaerephon *sp.	44	Zaire	0	0 (0–8.0)		0	0 (0–8.0)		0	0 (0–8.0)		0	0 (0–8.0)
	44	Sudan	0	0 (0–8.0)		0	0 (0–8.0)		0	0 (0–8.0)		0	0 (0–8.0)
* Mops *sp.	494	Zaire	6	1.2 (0.6–2.6)		6	1.2 (0.6–2.6)		1	0.2 (0.03–1.1)		1	0.2 (0.03–1.1)
	494	Sudan	2	0.4 (0.1–1.5)		2	0.4 (0.1–1.5)		0	0 (0–0.8)		0	0 (0–0.8)
*Nycteridae*													
* Nycteris *sp	22	Zaire	0	0 (0–14.9)		0	0 (0–14.9)		0	0 (0–14.9)		0	0 (0–14.9)
	22	Sudan	0	0 (0–14.9)		0	0 (0–14.9)		0	0 (0–14.9)		0	0 (0–14.9)
*Rhinolophidae*													
* Rhinolophus *sp.	86	Zaire	0	0 (0–4.3)		0	0 (0–4.3)		0	0 (0–4.3)		0	0 (0–4.3)
	86	Sudan	0	0 (0–4.3)		0	0 (0–4.3)		0	0 (0–4.3)		0	0 (0–4.3)
*Vespertilionidae*													
* Glauconycteris *sp.*	3	Zaire	0			0			0			0	
	3	Sudan	0			0			0			0	
* Kerivoula *sp.*	1	Zaire	0			0			0			0	
	1	Sudan	0			0			0			0	
Myotis bocagii*	3	Zaire	0			0			0			0	
	3	Sudan	0			0			0			0	
* Neoromicia *sp.*	5	Zaire	0			0			0			0	
	5	Sudan	0			0			0			0	
* Scotophilus *sp.	22	Zaire	0	0 (0–14.9)		0	0 (0–14.9)		0	0 (0–14.9)		0	0 (0–14.9)
	22	Sudan	0	0 (0–14.9)		0	0 (0–14.9)		0	0 (0–14.9)		0	0 (0–14.9)
*Pteropodidae*													
* Eidolon helvum*	480	Zaire	16	3.3 (2.1–5.4)		16	3.3 (2.1–5.4)		1	0.2 (0–1.2)		4	0.8 (0.3–2.1)
	480	Sudan	14	2.9 (1.7–4.8)		14	2.9 (1.7–4.8)		0	0 (0–0.8)		5	1.0 (0.4–2.4)
* Epomophorus *sp.	207	Zaire	1	0.5 (0.08–2.7)		1	0.5 (0.08–2.7)		0	0 (0–1.4)		0	0 (0–1.8)
	207	Sudan	0	0 (0–1.8)		0	0 (0–1.8)		0	0 (0–1.8)		0	0 (0–1.8)
* Epomops *sp.	280	Zaire	0	0 (0–1.4)		0	0 (0–1.4)		0	0 (0–1.4)		0	0 (0–1.4)
	280	Sudan	0	0 (0–1.4)		0	0 (0–1.4)		0	0 (0–1.4)		0	0 (0–1.4)
* Hypsignathus monstrosus*	185	Zaire	3	1.6 (0.6–4.7)		3	1.6 (0.6–4.7)		0	0 (0–2.0)		1	0.5 (0.05–3.0)
	185	Sudan	8	4.3 (2.2–8.3)		8	4.3 (2.2–8.3)		3	1.6 (0.6–4.7)		2	1.1(0.3–3.9)
* Lissonycteris angolensis*	84	Zaire	2	2.4 (0.7–8.3)		2	2.4 (0.7–8.3)		0	0 (0–4.4)		0	0 (0–4.4)
	84	Sudan	0	0 (0–4.4)		0	0 (0–4.4)		0	0 (0–4.4)		0	0 (0–4.4)
* Megaloglossus woermanni*	20	Zaire	0	0 (0–16.1)		0	0 (0–16.1)		0	0 (0–16.1)		0	0 (0–16.1)
	20	Sudan	0	0 (0–16.1)		0	0 (0–16.1)		0	0 (0–16.1)		0	0 (0–16.1)
* Micropteropus pusillus*	64	Zaire	0	0 (0–5.7)		0	0 (0–5.7)		0	0 (0–5.7)		0	0 (0–5.7)
	64	Sudan	1	1.6 (0.3–8.3)		1	1.6 (0.3–8.3)		0	0 (0–5.7)		0	0 (0–5.7)
* Myonycteris torquata*	56	Zaire	0	0 (0–6.4)		0	0 (0–6.4)		0	0 (0–6.4)		0	0 (0–6.4)
	56	Sudan	0	0 (0–6.4)		0	0 (0–6.4)		0	0 (0–6.4)		0	0 (0–6.4)
* Rousettus aegyptiacus*	359	Zaire	9	2.5 (1.3–4.7)		9	2.5 (1.3–4.7)		0	0 (0–1.1)		2	0.6 (0.2–2.0)
	359	Sudan	5	1.4 (0.6–3.2)		5	1.4 (0.6–3.2)		0	0 (0–1.1)		3	0.8 (0.3–2.4)
Scotonycteris zenkeri*	1	Zaire	0			0			0			0	
	1	Sudan	0			0			0			0	

*Percentages were not calculated because the number of samples collected was too low.

### Comparison of Zaire Ebola Virus Seroprevalence in Bats from Africa across Studies

For comparison, we compiled data regarding Zaire Ebola virus serology in bats of known species from previous studies (n = 4,493) and this study (n = 3,935; 46.7%) (Tables 5, 6). Data were available for 3,023 insectivorous bats of ≈30 species from 7 different families; 2,199 (72.7%) were from this study (Table 5). Insectivorous bat samples originated from Guinea, Cameroon, the DRC, and Gabon. Zaire Ebola virus reactivity has been observed only in *M. condylurus* bat samples from Gabon and *Mops* sp. bat samples from Cameroon. Data were available for 5,405 frugivorous bats of 17 species from 12 genera from West (Guinea, Ghana), West Central (Cameroon, Gabon, the Congo, the DRC), and East (Zambia) Africa (Table 6). No Zaire Ebola virus reactivity has been seen in blood samples from bat species *Casinycteris*, *Megaloglossus*, *Nanonycteris*, and *Scotonycteris*, but only a limited number of samples (n = 152) have been tested. Overall, blood samples from 8 frugivorous bat species have been found reactive with Zaire Ebola virus antigens. Blood samples from *E. helvum*, *H. monstrosus*, and *R. aegyptiacus* bats from several countries across Africa have been reported to be seropositive. Reactivity has been observed with samples from *E. gambianus* bats in Ghana (10.8%) and Guinea. Reactivity was observed with large sample sets from *E. franqueti* bats derived from Gabon and the Congo and a small sample set from Ghana but not Guinea, Cameroon, or the DRC. *M. pusillus* and *M. torquata* bats tested positive for Zaire Ebola virus antibodies in studies in which large sample sets were collected. Among *L. angolensis* bat samples, only those from Cameroon have tested positive for antibodies.

**Table 5:** Zaire Ebola virus antibodies in insectivorous bats from our research, Guinea, Cameroon, and the DRC, 2015–2017, and other published studies*

Family	Species	Country	Year of study (reference)	Test	No. tested	No. (%) positive†	Total, no. positive/tested (%)†
*Emballonuridae*	*Coleura afra*	Cameroon	2015–2017‡	Luminex	5	0–0 (0–0)	0/14 (0)
	*Saccolaimus peli*	DRC	1979–1980 (*26*)	IFA	9	0 (0)	
*Hipposideridae*	*Hipposideros* sp.	DRC	2015–2017‡	Luminex	157	0–0 (0–0)	0/1,395 (0)
	*Hipposideros* sp.	Cameroon	2015–2017‡	Luminex	837	0–0 (0–0)	
	*Hipposideros* sp.	DRC	1979–1980 (*26*)	IFA	69	0 (0)	
	*Hipposideros* sp.	Guinea	2015–2017‡	Luminex	288	0–0 (0–0)	
	*Hipposideros* sp.	Guinea	2014 (*11*)	ELISA	44	0 (0)	
*Miniopteridae*	*Miniopterus* sp.	Guinea	2015–2017‡	Luminex	27	0–0 (0–0)	0/234 (0)
	*Miniopterus* sp.	DRC	2015–2017‡	Luminex	205	0–0 (0–0)	
	*M. minor*	DRC	1995 (*27*)	ELISA	2	0 (0)	
*Molossidae*	*Chaerephon* sp.	Guinea	2015–2017‡	Luminex	44	0–0 (0–0)	0/401 (0)
	*C. pumilus*	Guinea	2014 (*11*)	ELISA	1	0 (0)	
	*C. ansorgei*	DRC	1995 (*27*)	ELISA	120	0 (0)	
	*C. major*	DRC	1979–1980 (*26*)	IFA	26	0 (0)	
	*C. pumilus*	DRC	1995 (*27*)	Elisa	210	0 (0)	
	*Mops* sp.	Guinea	2015–2017‡	Luminex	230	0–0 (0–0)	4–9/705 (0.6–1.3)
	*Mops* sp.	Cameroon	2015–2017‡	Luminex	264	1–6 (0.4–2.3)	
	*Mops* sp.	DRC	1979–1980 (*26*)	IFA	158	0 (0)	
	*Mops* sp.	DRC	1995 (*27*)	ELISA	28	0 (0)	
	*Mops condylurus*	Gabon	2003–2008 (*13*)	ELISA	24	3 (12.5)	
	*M. condylurus*	Guinea	2014 (*11*)	ELISA	1	0 (0)	
	*Myopterus whitleyi*	DRC	1995 (*27*)	ELISA	2	0 (0)	
*Nycteridae*	*Nycteris* sp.	Guinea	2015–2017‡	Luminex	15	0–0 (0–0)	0/43 (0)
	*Nycteris* sp.	Guinea	2014 (*11*)	ELISA	6	0 (0)	
	*Nycteris* sp.	Cameroon	2015–2017‡	Luminex	7	0–0 (0–0)	
	*Nycteris* sp.	DRC	1979–1980 (*26*)	IFA	14	0 (0)	
	*Nycteris hispida*	DRC	1995 (*27*)	ELISA	1	0 (0)	
*Rhinolophidae*	*Rhinolophus* sp.	Guinea	2015–2017‡	Luminex	26	0–0 (0–0)	0/86 (0)
	*Rhinolophus* sp.	DRC	2015–2017‡	Luminex	6	0–0 (0–0)	
	*Rhinolophus* sp.	Cameroon	2015–2017‡	Luminex	54	0–0 (0–0)	
*Vespertilionidae*	*Glauconycteris variegata*	Cameroon	2015–2017‡	Luminex	3	0–0 (0–0)	0/143 (0)
	*Chalinolobus* sp.	DRC	1979–1980 (*26*)	IFA	15	0 (0)	
	*Eptesicus* sp.	DRC	1979–1980 (*26*)	IFA	22	0 (0)	
	*Eptesicus tenuipinnis*	DRC	1995 (*27*)	ELISA	1	0 (0)	
	*Kerivoula* sp.	Guinea	2014 (*11*)	ELISA	1	0 (0)	
	*Kerivoula* sp.	Cameroon	2015–2017‡	Luminex	1	0–0 (0–0)	
	*Myotis bocagii*	Cameroon	2015–2017‡	Luminex	3	0–0 (0–0)	
	*M. bocagii*	DRC	1995 (*27*)	ELISA	22	0 (0)	
	*M. bocagii*	DRC	1979–1980 (*26*)	IFA	17	0 (0)	
	*Neoromicia* sp.	Cameroon	2015–2017‡	Luminex	5	0–0 (0–0)	
	*Pipistrellus nanus*	DRC	1995 (*27*)	ELISA	2	0 (0)	
	*Scotophilus nux*	Cameroon	2015–2017‡	Luminex	6	0–0 (0–0)	
	*Scotophilus leucogaster*	Guinea	2015–2017‡	Luminex	15	0–0 (0–0)	
	*Scotophilus nigrita*	Guinea	2015–2017‡	Luminex	1	0–0 (0–0)	
	*Scotophilus dinganii*	DRC	1995 (*27*)	ELISA	19	0 (0)	
	*Scotophilus* sp.	DRC	1979–1980 (*26*)	IFA	10	0 (0)	
Total							4–9/3,023 (0.13–0.30)

*DRC, the Democratic Republic of the Congo; IFA, immunofluorescence assay. †For data from cited studies, the number of positive samples reported in the original study is indicated. For our results, we show the range in the number of samples simultaneously reactive with glycoprotein and nucleoprotein of Zaire Ebola virus on the basis of 4 different statistical methods used to determine cutoff values. ‡This study.

**Table 6:** Zaire Ebola virus antibodies in frugivorous (*Pteropodidae* family) bats from our research, Guinea, Cameroon, and the DRC, 2015–2017, and published studies*

Species	Country	Year of study (reference)	Test	No. tested	No. (%) positive	Total, no. positive/tested (%)
*Casinycteris ophiodon*	Guinea	2014 (*11*)	ELISA	1	0	0/20
*Casinycteris argynnis*	Gabon, Congo	2003–2008 (*13*)	ELISA	18	0	
*C. argynnis*	DRC	1995 (*27*)	ELISA	1	0	
*Eidolon helvum*†	Guinea	2014 (*11*)	ELISA	6	0	21–36/1,551 (1.4–2.3)
	Guinea	2015–2017‡	Luminex	17	0–3 (0–17.6)	
	Ghana	2008 (*14*)	IFA	262	1 (0.39)	
	Cameroon	2015–2017‡	Luminex	158	1–9 (0.6–5.7)	
	Gabon, Congo	2003–2008 (*13*)	ELISA	49	0	
	DRC	1979–1980 (*26*)	IFA	6	0	
	DRC	2015–2017‡	Luminex	305	0–4 (0–1.3)	
	Zambia	2006–2013 (*16*)	ELISA	748	19 (2.55)	
*Epomophorus gambianus*	Guinea	2015–2017‡	Luminex	191	0–1 (0–0.5)	4–5/244 (1.6–2.0)
	Ghana	2007 (*15*)	ELISA	37	4 (10.82)	
*Epomophorus wahlbergi*	Cameroon	2015–2017‡	Luminex	16	0–0 (0–0)	
*Epomops buettikoferi*	Guinea	2014 (*11*)	ELISA	17	0	47/1,269 (3.7)
	Guinea	2015–2017‡	Luminex	4	0–0 (0–0)	
*Epomops franqueti*	Ghana	2007 (*15*)	ELISA	27	3 (11.2)	
	Cameroon	2015–2017‡	Luminex	256	0–0 (0–0)	
	Gabon, Congo	2001–2005 (*6*)	ELISA	117	8 (6.8)	
	Gabon, Congo	2003–2008 (*13*)	ELISA	805	36 (4.5)	
	DRC	2015–2017‡	Luminex	20	0–0 (0–0)	
	DRC	1979–1980 (*26*)	IFA	21	0	
	DRC	1995 (*27*)	ELISA	2	0	
*Hypsygnathus monstrosus*	Guinea	2015–2017‡	Luminex	8	0–0 (0–0)	15–18/347 (4.3–5.2)
	Guinea	2014 (*13*)	ELISA	1	0	
	Ghana	2008 (*14*)	IFA	3	0	
	Ghana	2007 (*15*)	ELISA	16	2 (12.5)	
	Cameroon	2015–2017‡	Luminex	176	0–3 (0–1.7)	
	Gabon, Congo	2001–2005 (*6*)	ELISA	17	4 (23.5)	
	Gabon, Congo	2003–2008 (*13*)	ELISA	125	9 (7.2)	
	DRC	2015–2017‡	Luminex	1	0–0 (0–0)	
*Lissonycteris angolensis*	Guinea	2014 (*11*)	ELISA	45	0	0–2/129 (0–1.6)
	Guinea	2015–2017‡	Luminex	32	0–0 (0–0)	
	DRC	2015–2017‡	Luminex	22	0–0 (0–0)	
	Cameroon	2015–2017‡	Luminex	30	0–2 (0–6.7)	
*Megaloglossus azagnyi*	Guinea	2014 (*11*)	ELISA	3	0	0/110
*Megaloglossus woermanni*	Cameroon	2015–2017‡	Luminex	19	0–0 (0–0)	
	Gabon, Congo	2003–2008 (*13*)	ELISA	49	0	
	DRC	2015–2017‡	Luminex	1	0–0 (0–0)	
	DRC	1995 (*27*)	ELISA	38	0	
*Micropteropus pusillus*	Guinea	2015–2017‡	Luminex	18	0–0 (0–0)	4/339 (1.2)
	Cameroon	2015–2017‡	Luminex	2	0–0 (0–0)	
	Gabon, Congo	2003–2008 (*13*)	ELISA	197	4 (2.04)	
	DRC	2015–2017‡	Luminex	44	0–0 (0–0)	
	DRC	1995 (*27*)	ELISA	78	0	
*Myonycteris leptodon*	Guinea	2014 (*11*)	ELISA	21	0	23–27/708 (3.2–3.8)
*Myonycteris torquata*	Cameroon	2015–2017‡	Luminex	21	0–0 (0–0)	
	Gabon, Congo	2001–2005 (*6*)	ELISA	58	4 (6.9)	
	Gabon, Congo	2003–2008 (*13*)	ELISA	573	19 (3.32)	
	DRC	2015–2017‡	Luminex	35	0–0 (0–0)	
*Nanonycteris veldkampii*	Guinea	2014 (*11*)	ELISA	17	0	0/21
	Ghana	2007 (*15*)	ELISA	4	0	
*Rousettus aegyptiacus*	Guinea	2015–2017‡	Luminex	228	0–1 (0–0.4)	24–33/666 (3.6–5.0)
	Cameroon	2015–2017‡	Luminex	131	0–8 (0–6.1)	
	Gabon, Congo	2003–2008 (*13*)	ELISA	307	24 (7.8)	
*Scotonycteris zenkeri*	Cameroon	2015–2017‡	Luminex	1	0–0 (0–0)	0–0/1 (0–0)
Total						138–172/5,405 (2.55–3.18)

*DRC, the Democratic Republic of the Congo; IFA, immunofluorescence assay. †For cited studies, the number of positive samples reported in the original study is indicated. For our results, we show the range in the number of samples simultaneously reactive with glycoprotein and nucleoprotein of Zaire Ebola virus on the basis of 4 different statistical methods used to determine cutoff values. ‡This study.

### RT-PCR Screening for Zaire Ebola Virus RNA

We screened 665 samples from the DRC (n = 193), Cameroon (n = 399), and Guinea (n = 73) by RT-PCR for the presence of Zaire Ebola virus RNA. Of the 294 samples originating from bats previously documented to carry Zaire Ebola virus RNA (*6*) (i.e., *H. monstrosus* [132 from Cameroon, 1 from the DRC], *M. torquata* [20 from Cameroon, 25 from the DRC], and *E. franqueti* [116 from Cameroon]), all were negative for Zaire Ebola virus RNA. Of the 371 samples from bat species *E. helvum* (58 from Cameroon, 165 from the DRC, 3 from Guinea), *L. angolensis* (8 from Cameroon, 4 from Guinea), *M. pusillus* (2 from the DRC, 1 from Guinea), *R. aegyptiacus* (45 from Cameroon, 40 from Guinea), *E. gambianus* (25 from Guinea), and *Mops* sp. (20 from Cameroon), all were negative for Zaire Ebola virus RNA.

## Discussion

To clarify the role of bats in Ebola virus ecology and identify where the virus circulates between outbreaks, we tested >4,000 bats, almost doubling the total number of samples tested in all previous studies in Africa (*5*–*7*,*42*). We provided data on bats from Cameroon, added to the existing data on bats from Guinea and the DRC, and substantially increased the data available on insectivorous bats. We tested samples with the same assay, enabling comparison across species and countries. We used different statistical methods to determine positive sample numbers and expressed the proportion of reactive samples as a range on the basis of the different cutoff values proposed by those methods. As has been done in studies of human Zaire Ebola virus survivors (*28*,*43*), we defined Zaire and Sudan Ebola virus positivity as the presence of antibodies to both nucleoprotein and glycoprotein. As such, we estimated that 2–37 (0.05%–0.92%) bats were seropositive for Zaire Ebola virus and 0–30 (0%–0.75%) bats were seropositive for Sudan Ebola virus (Table 3). Among insectivorous bats, we observed Zaire and Sudan Ebola virus antibodies only in *Mops* sp. bats, an observation that has previously been observed (*13*). We provided information on insectivorous *Miniopterus* and *Rhinolophus* bats and extended knowledge on *Mops* and *Hipposideros* bats; all 1,200 *Hipposideros* samples were seronegative. We confirmed the presence of Zaire Ebola virus antibodies in only 1 of 3 frugivorous species in which Zaire Ebola virus RNA has been reported, that is, in *H. monstrosus* but not *E. franqueti* or *M. torquata* bats (*6*). However, this result might have been influenced by sample size, test used, and interpretation criteria. We confirmed antibodies in *E. helvum* bats and showed that Zaire Ebola virus antibodies are widespread among this species across Africa: Ghana and Zambia, and with our data, also Cameroon, Guinea, and the DRC (*13*,*14*,*16*). We confirmed antibodies in *R. aegyptiacus* bats from Cameroon and Guinea, in agreement with previous findings in these bats from the Congo and Gabon (*13*). For *E. gambianus* bats from Ghana, we also observed Zaire Ebola virus reactivity of samples from this species in Guinea (*15*). In contrast with a previous study, we observed Sudan Ebola virus antibodies (not Zaire Ebola virus antibodies) in *M. pusillus* bats (*13*). We also identified Zaire Ebola virus antibodies in *L. angolensis* bats from Cameroon, although only when using less stringent cutoff calculations.

When combining data from previous Zaire Ebola virus seroprevalence studies in bats with data from our study, only 1 insectivorous bat species (*Mops* sp.) and 8 frugivorous bat species (*E. helvum*, *E. gambianus*, *E. franqueti*, *H. monstrosus*, *L. angolensis*, *M. pusillus*, *M. torquata*, *R. aegyptiacus*) exhibited Zaire Ebola virus antibodies (*13*–*16*). As seen in bat samples from Zambia, we observed in this study Sudan Ebola virus antibodies in *E. helvum* bats from Guinea, Cameroon, and the DRC, suggesting that Zaire and Sudan Ebola viruses co-circulate and could be widespread among this species. However, only 1 other study has tested for Ebola viruses other than Zaire Ebola virus in *E. helvum* bats (*16*). In our study, we also observed Sudan Ebola virus antibodies in *Mops* sp., *H. monstrosus*, and *R. aegyptiacus* bats in Cameroon. Almost all samples were positive for either Zaire or Sudan Ebola virus but not for both.

Despite the presence of Ebola virus antibodies, the role of bats as reservoir species remains unclear because viral RNA detection is rare. In only 1 study Zaire Ebola virus RNA was amplified in a few bats (*6*). Thus, antibodies might reflect previous acute infection with viral clearance. Unlike inoculations with Marburg virus (*44*–*46*), experimental inoculation of *R. aegyptiacus* bats with Zaire Ebola virus leads to antibody development but infrequent or absent detection of viral RNA or shedding (*44*,*47*). *R. aegyptiacus* bats are therefore able to clear Zaire Ebola virus after a short infectious period without viral shedding and with little or no transmission. No antibodies or viral RNA were detected in noninoculated bats housed with experimentally Zaire Ebola virus–infected bats (*44*). Whether this low level of infectiousness also occurs for other bat species that carry Ebola virus antibodies remains to be determined. Zaire Ebola virus was experimentally inoculated in other bat species (*M. condylurus*, *Chaerephon pumilus*, and *Epomophorus wahlbergi*) in only 1 study; virus replication was seen in all species, and fecal shedding was seen in *E. wahlbergi* bats (*48*). *R. aegyptiacus* bats experimentally infected with Marburg virus were shown to develop antibodies that protect against reinfection (*49*). Long-term survival with Zaire Ebola virus antibodies has been reported with *E. helvum* bats from Ghana but without information on protection (*14*). Among insectivorous bats, the presence of Ebola virus antibodies in only *Mops* sp. is striking, suggesting higher exposure or susceptibility compared with other insectivorous bats.

In conclusion, we demonstrated higher rates of Ebola virus antibodies in frugivorous than in insectivorous bats. The total number of frugivorous species shown to be Zaire Ebola virus seropositive has increased to 8, and 1 insectivorous bat species (*Mops* sp.) was confirmed to be seropositive. Zaire and Sudan Ebola viruses circulate in different species across Africa, with potential co-circulation of both viruses in some species. Although we have data on >8,000 bats from >40 species, this sample size is small, given the high numbers of bats that constitute colonies. This study illustrates the complexity of tracking the animal reservoir of Ebola viruses, not only because sampling of wild bats without performing euthanasia is difficult and time-consuming but also because of the absence of a reference standard for serologic tests. To clarify the significance of Ebola virus antibodies, documenting the extent to which viral RNA and shedding can be detected in species with antibodies is crucial for predicting and controlling the risk for new outbreaks. Efforts must continue not only to sample bats but also other animals to elucidate where the virus circulates in wildlife.

Technical Appendix Description of serum sample dilution testing, mean fluorescence intensity cutoff values, and Ebola virus antibody cross reactivity.
