# Supplementary material for: Survey of Ebola Viruses in Frugivorous and Insectivorous Bats in Guinea, Cameroon, and the Democratic Republic of the Congo, 2015–2017
Source: Emerg Infect Dis. 2018 Dec;24(12):2228–40. doi: 10.3201/eid2412.180740 (PMC6256401; doi:10.3201/eid2412.180740)
Supplement: Technical Appendix — Description of serum sample dilution testing, mean fluorescence intensity cutoff values, and Ebola virus antibody cross reactivity. [file 18-0740-Techapp-s1.pdf]

Article DOI: https://doi.org/10.3201/eid2412.180740

Survey of Ebola Viruses in Frugivorous and Insectivorous Bats in Guinea, Cameroon, and the Democratic Republic of the Congo, 2015–2017

Technical Appendix

Adaptation of Serologic Assay for Screening of Bats

In most studies on Ebola virus serology of bats, plasma or serum samples diluted in the range of 1:50 through 1:400 were tested (*1–5*). To test adequacy of these dilutions, we first titrated plasma samples obtained from bats living in Wilhelma Zoo and Botanical Garden (Stuttgart, Germany) and wild bats in Cameroon. We performed dilutions from 1:200 through 1:2,000 with European bats *Pteropus giganteus* (n = 4) and *Rousettus aegyptiacus* (n = 4) and tested these dilutions with antigens on our panel. At each of the 5 dilutions tested, the measured mean fluorescence intensities (MFIs) were <500 for all Ebola virus antigens; when plasma samples were diluted 1:2,000, MFIs were even lower (<200). Next, we tested 20 bat samples from Cameroon diluted 1:200, 1:400, and 1:800 on the same antigens. For these samples, MFIs as high as 6,000 and 12,000 were observed at dilutions 1:800 and 1:200, respectively. If European bat samples are considered Ebola virus negative and used to calculate cutoff values, Ebola virus antibody prevalence could reach up to 8–for Sudan Ebola virus glycoprotein at dilution 1:200. Even with our stringent nucleoprotein plus glycoprotein positivity criteria, the proportion of Zaire Ebola virus–positive samples was 66% for dilution 1:200 and 22% for dilution 1:800. Therefore, we tested all bat samples at a dilution of 1:2,000 to have a stricter significance threshold and to identify the samples that are most likely true positives.

References

1. Pourrut X, Souris M, Towner JS, Rollin PE, Nichol ST, Gonzalez JP, et al. Large serological survey showing cocirculation of Ebola and Marburg viruses in Gabonese bat populations, and a high

seroprevalence of both viruses in *Rousettus aegyptiacus*. BMC Infect Dis. 2009;9:159. PubMed http://dx.doi.org/10.1186/1471-2334-9-159

2. Leirs H, Mills JN, Krebs JW, Childs JE, Akaibe D, Woollen N, et al. Search for the Ebola virus reservoir in Kikwit, Democratic Republic of the Congo: reflections on a vertebrate collection. J Infect Dis. 1999;179(Suppl 1):S155–63. PubMed http://dx.doi.org/10.1086/514299

3. Hayman DT, Emmerich P, Yu M, Wang LF, Suu-Ire R, Fooks AR, et al. Long-term survival of an urban fruit bat seropositive for Ebola and Lagos bat viruses. PLoS One. 2010;5:e11978. PubMed http://dx.doi.org/10.1371/journal.pone.0011978

4. Hayman DT, Yu M, Crameri G, Wang LF, Suu-Ire R, Wood JL, et al. Ebola virus antibodies in fruit bats, Ghana, West Africa. Emerg Infect Dis. 2012;18:1207–9. PubMed http://dx.doi.org/10.3201/eid1807.111654

5. Ogawa H, Miyamoto H, Nakayama E, Yoshida R, Nakamura I, Sawa H, et al. Seroepidemiological prevalence of multiple species of filoviruses in fruit bats (*Eidolon helvum*) migrating in Africa. J Infect Dis. 2015;212(Suppl 2):S101–8. PubMed http://dx.doi.org/10.1093/infdis/jiv063

Technical Appendix Table 1. Mean fluorescence intensity cutoff values for different virus antigens, by Ebola virus lineage and statistical method used to determine cutoff, Guinea, Cameroon, and the Democratic Republic of the Congo, 2015–2017

	Statistical method			
Ebola virus lineage, antigen	Mean + 4×SD	Change-point	Binomial	Exponential
Zaire				
Nucleoprotein	71	382	165	76
Glycoprotein Kissidougou*	128	406	1,231	492
Glycoprotein Mayinga†	307	484	1,475	566
Viral protein 40	75	324	155	76
Sudan				
Nucleoprotein	131	608	265	111
Glycoprotein	251	929	2,831	1,089
Viral protein 40	88	294	240	120
Bundibunyo				
Glycoprotein	98	389	714	296
Viral protein 40	363	398	162	369
Reston				
Glycoprotein	249	147	163	70

*Glycoprotein recombinant derived from Zaire Ebola virus strain Kissidougou from West Africa in 2014.
†Glycoprotein recombinant derived from Zaire Ebola virus strain Mayinga from the Democratic Republic of the Congo in 1976.

Technical Appendix Table 2. Cross-reactivity of bat antibodies to Ebola virus antigens of different lineages, by statistical method used to determine cutoff, Guinea, Cameroon, and the Democratic Republic of the Congo, 2015–2017*

	Statistical method, no./total (%)			
Ebola virus antigen	Mean + 4×SD	Change-point	Binomial	Exponential
NP	6/151 (3.98)	1/24 (4.17)	1/66 (1.52)	7/158 (4.44)
GP	443/578 (76.7)	131/223 (58.8)	21/65 (32.3)	86/236 (36.5)
VP	35/169 (20.72)	7/38 (18.4)	17/50 (34.0)	40/122 (32.8)

*Values are expressed as number of samples reactive with >1 Ebola virus antigen over total number of samples reactive with at least 1 NP, GP, or VP Ebola virus antigen. GP, glycoprotein; NP, nucleoprotein; VP, viral protein 40.
